# Supplementary material for: Stepwise maturation of the peptidyl transferase region of human mitoribosomes
Source: Nat Commun. 2021 Jun 16;12:3671. doi: 10.1038/s41467-021-23811-8 (PMC8208988; doi:10.1038/s41467-021-23811-8)
Supplement: Supplementary file 1 — Supplementary Information [file 41467_2021_23811_MOESM1_ESM.pdf]

Supplementary Information for

Stepwise maturation of the peptidyl transferase region of human mitoribosomes

Tea Lenarčič[1†], Mateusz Jaskolowski[1†], Marc Leibundgut[1], Alain Scaiola[1], Tanja Schönhut[1], Martin Saurer[1], Richard G. Lee[2,3], Oliver Rackham[2,3,4,5], Aleksandra Filipovska[2,3,5] & Nenad Ban[1*]

[1]Department of Biology, Institute of Molecular Biology and Biophysics, ETH Zurich, Otto-Stern-Weg 5, CH-8093 Zurich, Switzerland

[2]Harry Perkins Institute of Medical Research, QEII Medical Centre, University of Western Australia, Western Australia 6009, Australia

[3]ARC Centre of Excellence in Synthetic Biology, QEII Medical Centre, University of Western Australia, Western Australia 6009, Australia

[4]Curtin Health Innovation Research Institute and Curtin Medical School, Curtin University, Bentley, Western Australia 6102, Australia

[5]Telethon Kids Institute, Northern Entrance, Perth Children's Hospital, 15 Hospital Avenue, Nedlands, Western Australia 6009, Australia

[†]These authors contributed equally to this work.

*Correspondence should be addressed to N.B.

Supplementary Figures

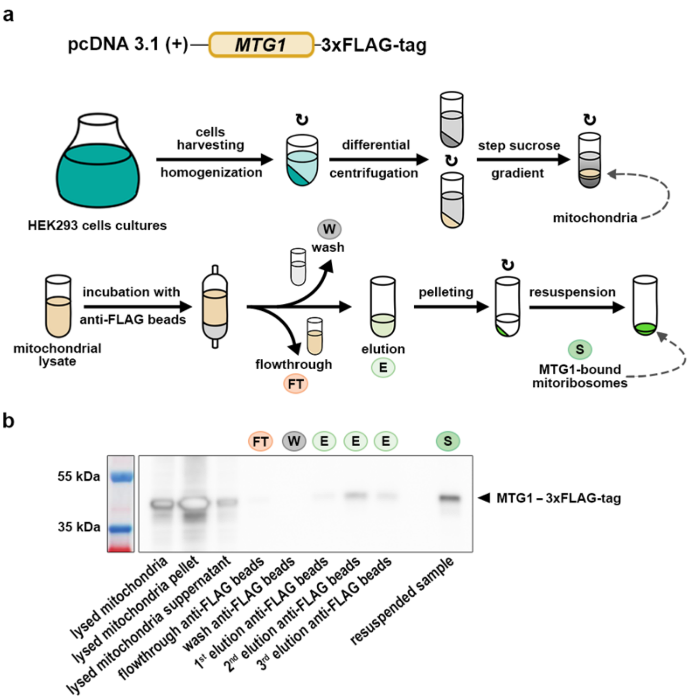

Supplementary Figure 1. 3x-FLAG tagged MTG1 co-pellets with the mitoribosomal particles. (**a**) Schematic representation of the mitochondria preparation, followed by the purification of mitoribosomes *via* C-terminally 3x-FLAG-tagged MTG1**.** Curved arrows indicate an (ultra)centrifugation step. (**b**) Western Blot analysis of two independent transfection and purification procedures using antibodies against the 3xFLAG tag yielded similar results. Source data are provided as a Source Data file.

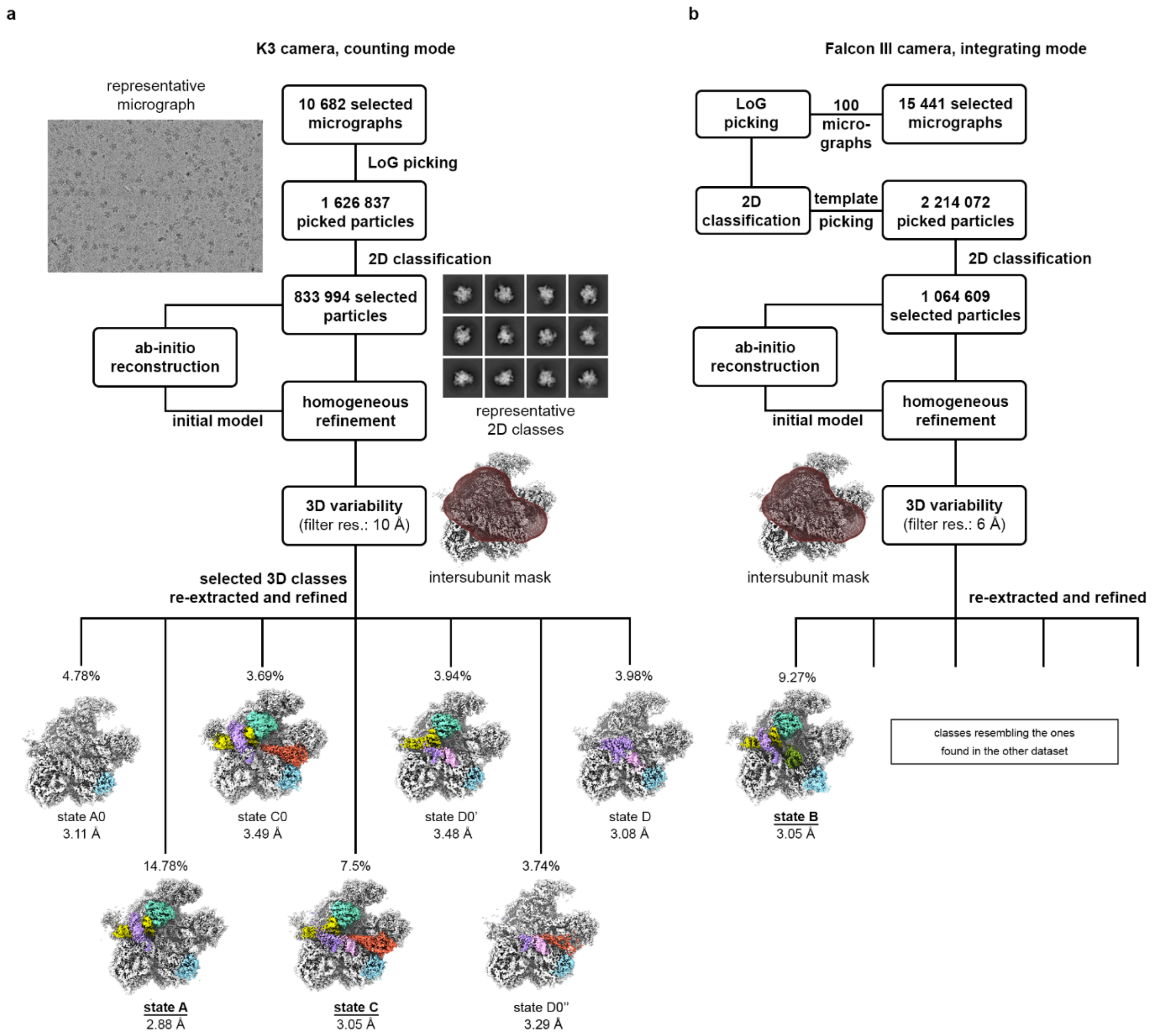

Supplementary Figure 2. Data processing of the purified particles bound to the tagged MTG1 assembly factor. Data processing schemes for both datasets collected from the particles obtained by affinity purification of C-terminally tagged MTG1 assembly factor. Both datasets, collected using K3 (**a**) and Falcon IIIEC (**b**) cameras, were processed independently. Cryo-EM maps of selected 3D classes with density of assembly factors as well as key rRNA regions color-coded as in Fig. 1 are shown.

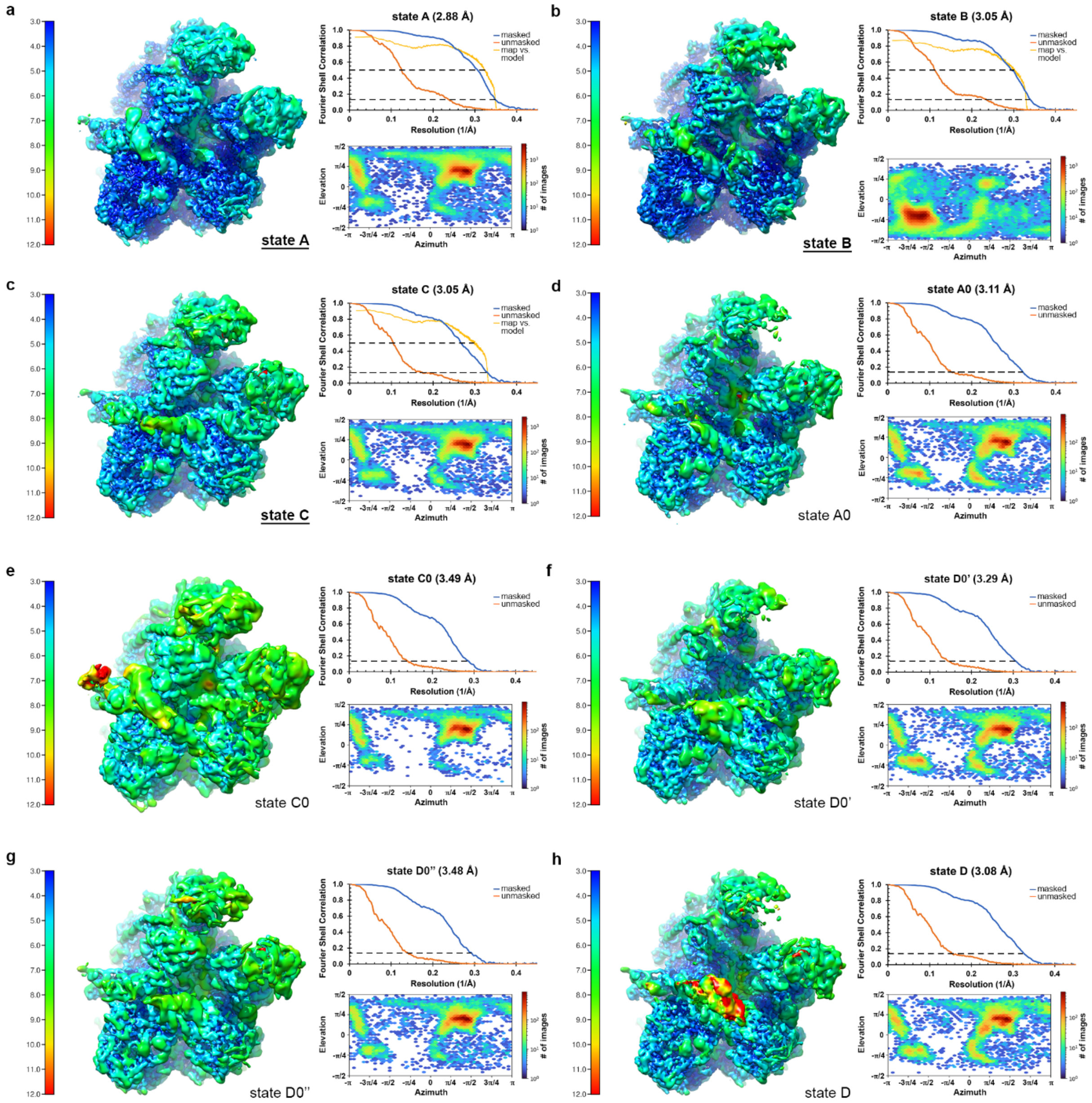

Supplementary Figure 3. Cryo-EM data statistics. Cryo-EM maps, colored according to the local resolution with scale bars presented on the left of each panel, are shown for state A (**a**), state B (**b**), state C (**c**), state A0 (**d**), state C0 (**e**), state D0' (**f**), state D0'' (**g**) and state D (**h**). Each panel contains the FSC curve on the top right and the particles angular distribution graph on the bottom right.

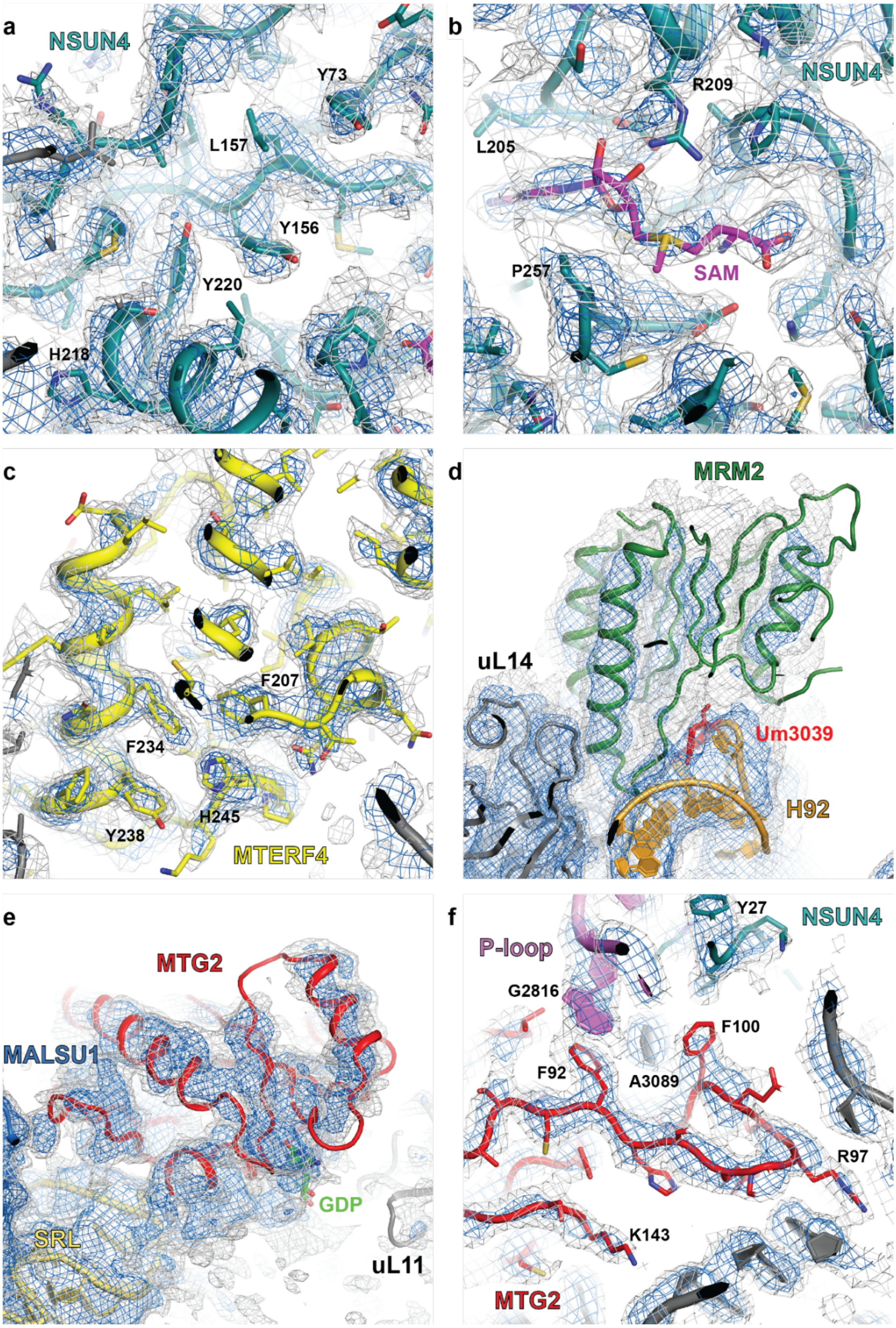

Supplementary Figure 4. Examples of electron density maps in the areas of the bound maturation factors. Overview of the EM density for (**a**) NSUN4, (**b**) the NSUN4 S-adenosyl-methionine cofactor, (**c**) MTERF4, (**d**) MRM2, (**e**) the G domain of MTG2 and (**f**) the N-terminal Obg domain of MTG2 and NSUN4 interacting with the P loop.

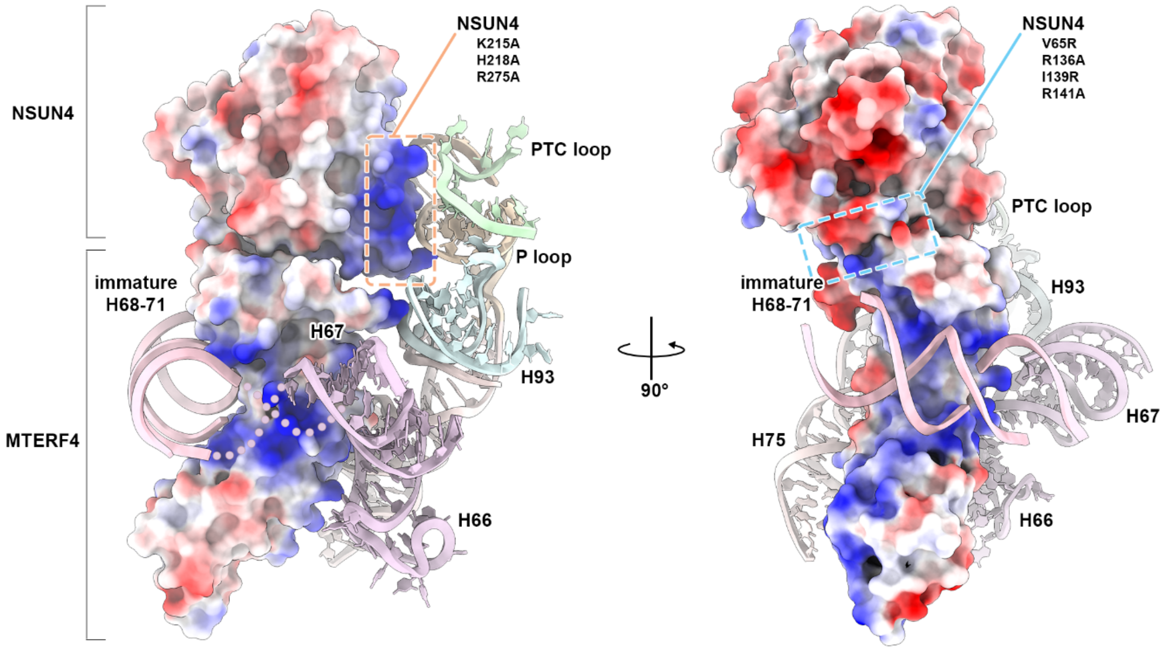

Supplementary Figure 5. NSUN4 binding to key elements of the PTC rRNA region and MTERF4 stabilizing a distinct immature conformation of H68-71. Atomic models of NSUN4–MTERF4 are shown in surface representation and colored according to their electrostatic potential. Selected rRNA elements that interact with NSUN4–MTERF4 are shown in cartoon and are labeled individually. NSUN4–MTERF4 is shown as viewed from the GTPase associated center (left) and from intersubunit space (right). Regions of introduced NSUN4 mutations are indicated with dashed line boxes.

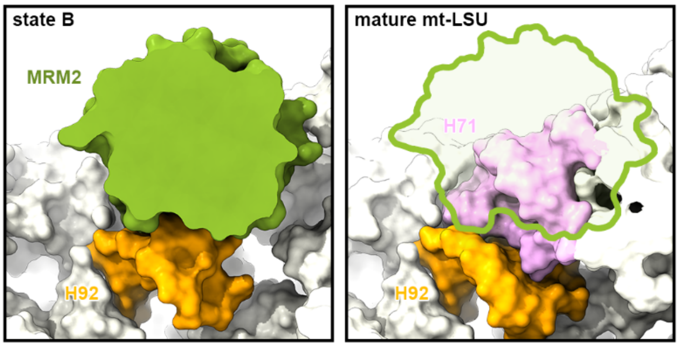

Supplementary Figure 6. MRM2 methylates U3039 in the A-loop before maturation of H71. Cross-sections of the atomic models of state B (left) and the mature mt-LSU (right, PDB: 6ZM6 [http://doi.org/10.2210/pdb6ZM6/pdb]) are shown as viewed from the CP. The MRM2 methyltransferase, H92 (A loop) and H71 are labeled and colored individually. On the right panel, the green outline represents superposed MRM2 to visualize steric hindrance of the assembly factor with the mature H71.

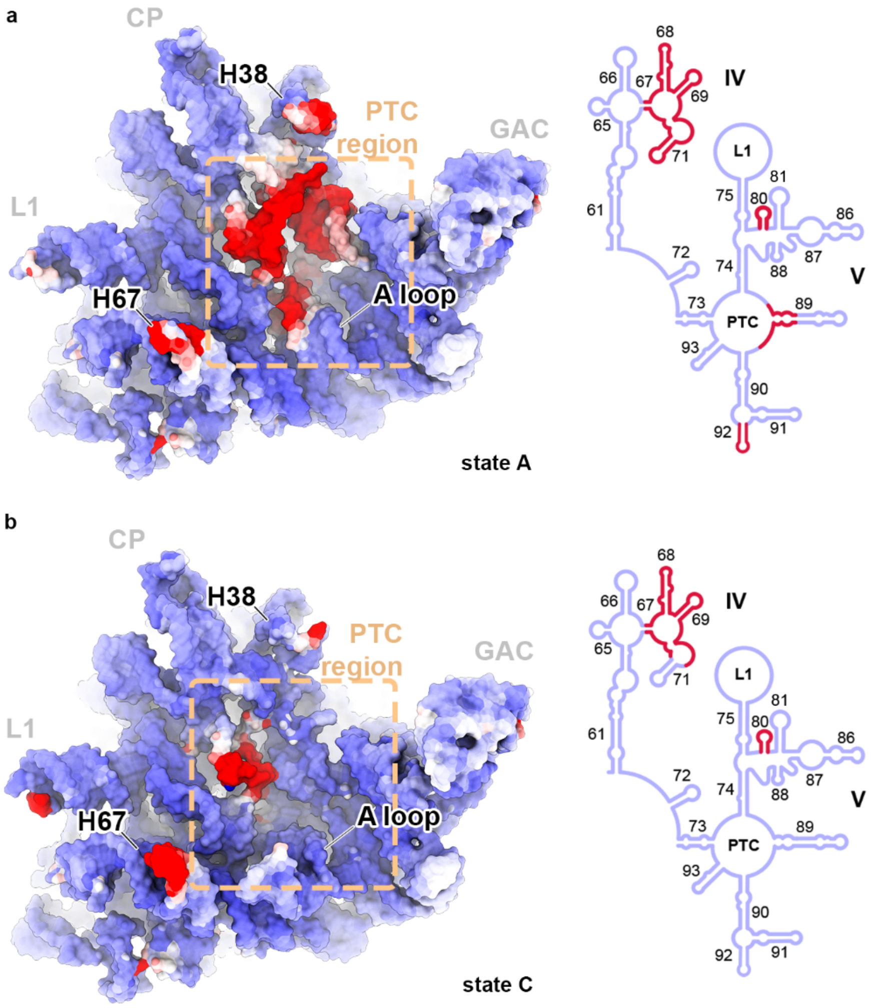

Supplementary Figure 7. Stepwise maturation of the key rRNA regions of the mt-LSU. The 16S rRNA atomic models of states A (**a**) and C (**b**) are shown in surface representation and colored according to the root-mean-square deviation (RMSD) compared to the mature 16S rRNA (PDB: 6ZM6 [http://doi.org/10.2210/pdb6ZM6/pdb]). RMSD values range from 0 Å (blue) to 8 Å and above (red). Corresponding 2D diagrams of 16S rRNA domains IV and V are shown on the right of each panel and colored according to their respective atomic model.

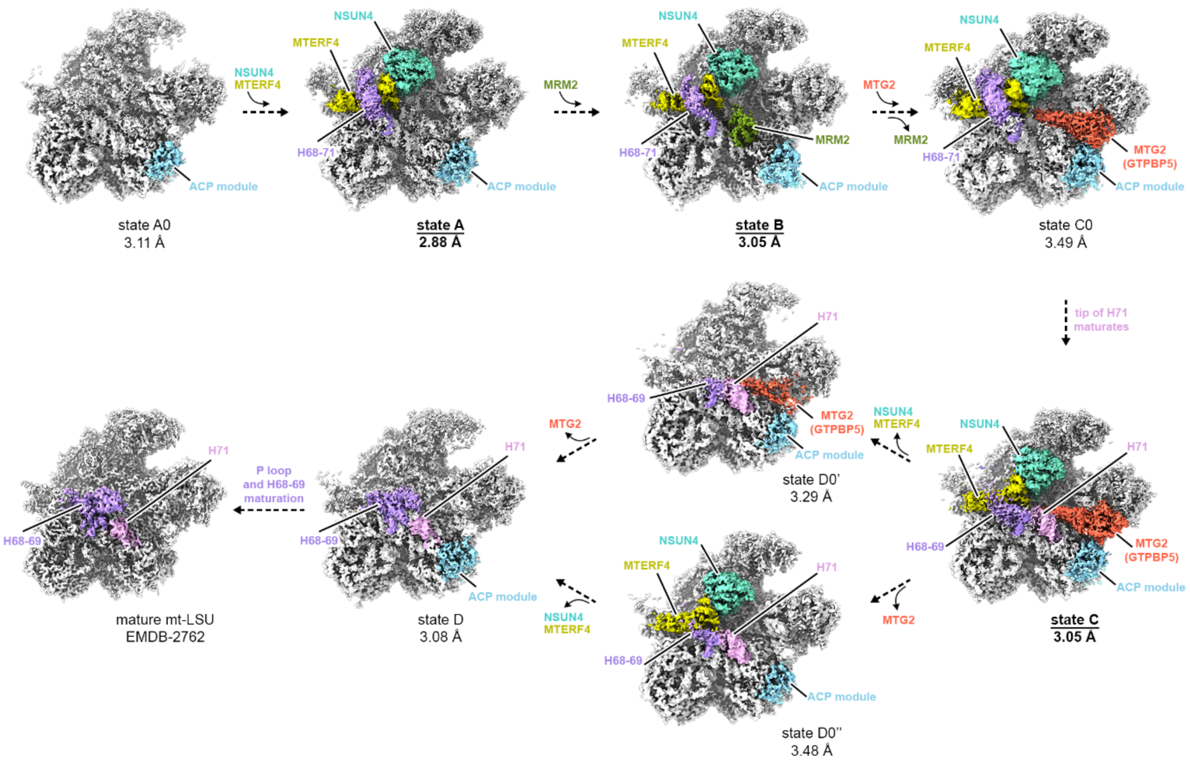

Supplementary Figure 8. Stepwise maturation of the mt-LSU including all obtained cryo-EM maps. The pathway of mt-LSU maturation is proposed based on all obtained cryo-EM maps. The densities corresponding to the assembly factors and important rRNA fragments are colored and labeled individually. The ACP module includes MALSU1, L0R8F8 and mt-ACP.

Supplementary Table 1. Cryo-EM data collection, refinement and validation statistics.

	**State A** MTERF4-NSUN4 + ACP module	**State B** MTERF4-NSUN4-MRM2 + ACP module	**State C** MTERF4-NSUN4-MTG2 + ACP module
EMDB code	EMD-12845	EMD-12846	EMD-12847
PDB code	7ODR	7ODS	7ODT
**Data collection and processing**			
Camera	K3	Falcon IIIEC	K3
Magnification	81,000 (nominal)	75,000 (nominal)	81,000 (nominal)
Voltage (kV)	300		
Electron exposure (e^{-}/Å^2)	60		
Defocus range (µm)	0.6-3.0		
Pixel size (Å)	1.06 (super-res. pix. at 0.53 Å/pix.)	1.087	1.06 (super-res. pix. at 0.53 Å/pix.)
Initial particle images (no.)	833,994	1,064,609	833,994
Final particle images (no.)	123,285	114,557	62,565
Map resolution at FSC=0.143 (Å)	2.9	3.1	3.1
**Structure refinement in PHENIX 1.19.1**			
Model resolution at FSC=0.5 (Å)	3.1	3.3	3.4
CC_{mask}	0.80	0.76	0.79
Map sharpening B factor (Å^2)	– 64.1	– 97.9	– 54.6
Model composition			
Non-hydrogen atoms	107,464	108,339	110,473
Protein residues	9,244	9,371	9,585
RNA residues	1,520	1,518	1,526
Ligands: Mg^{2+} / K^{+} / Zn^{2+} / [Fe_2-S_2] / C10-PPT / SAM / GDP	97 / 2 / 2 / 1 / 1 / 1 / -	88 / 8 / 2 / 1 / 1 / 1 / -	95 / 10 / 2 / 1 / 1 / 1 / 1
B factors min/max/mean (Å^2)			
Protein	14/117/54	22/253/92	27/238/98
RNA	9/173/50	20/369/74	30/311/76
Ligand	12/101/49	20/186/84	23/186/88
R.m.s. deviations			
Bond lengths (Å)	0.002	0.002	0.002
Bond angles (°)	0.404	0.397	0.405
**Validation**			
MolProbity score	1.3	1.3	1.3
Clashscore	4.9	5.0	5.2
Poor rotamers (%)	0.4	0.5	0.5
Protein			
EM Ringer score	3.3	2.4	2.8
Ramachandran plot			
Favored (%)	98.9	98.8	98.8
Allowed (%)	1.1	1.2	1.2
Disallowed (%)	0.0	0.0	0.0
RNA			
Pucker outliers (%)	0.1	0.5	0.5
Bond outliers (%)	0.0	0.0	0.0
Angle outliers (%)	0.0	0.0	0.0
Suite outliers (%)	17.0	18.0	17.8
