# Supplementary material for: Stepwise maturation of the peptidyl transferase region of human mitoribosomes
Source: Nat Commun. 2021 Jun 16;12:3671. doi: 10.1038/s41467-021-23811-8 (PMC8208988; doi:10.1038/s41467-021-23811-8)
Supplement: Supplementary file 2 — Reporting Summary [file 41467_2021_23811_MOESM2_ESM.pdf]

nature research

Corresponding author(s): Nenad Ban

Last updated by author(s): Apr 2, 2021

Reporting Summary

Nature Research wishes to improve the reproducibility of the work that we publish. This form provides structure for consistency and transparency in reporting. For further information on Nature Research policies, see our Editorial Policies and the Editorial Policy Checklist.

Statistics

For all statistical analyses, confirm that the following items are present in the figure legend, table legend, main text, or Methods section.

n/a	Confirmed	
☐	☒	The exact sample size (*n*) for each experimental group/condition, given as a discrete number and unit of measurement
☐	☒	A statement on whether measurements were taken from distinct samples or whether the same sample was measured repeatedly
☒	☐	The statistical test(s) used AND whether they are one- or two-sided<br>*Only common tests should be described solely by name; describe more complex techniques in the Methods section.*
☒	☐	A description of all covariates tested
☒	☐	A description of any assumptions or corrections, such as tests of normality and adjustment for multiple comparisons
☒	☐	A full description of the statistical parameters including central tendency (e.g. means) or other basic estimates (e.g. regression coefficient) AND variation (e.g. standard deviation) or associated estimates of uncertainty (e.g. confidence intervals)
☒	☐	For null hypothesis testing, the test statistic (e.g. *F*, *t*, *r*) with confidence intervals, effect sizes, degrees of freedom and *P* value noted<br>*Give P values as exact values whenever suitable.*
☒	☐	For Bayesian analysis, information on the choice of priors and Markov chain Monte Carlo settings
☒	☐	For hierarchical and complex designs, identification of the appropriate level for tests and full reporting of outcomes
☒	☐	Estimates of effect sizes (e.g. Cohen's *d*, Pearson's *r*), indicating how they were calculated

Our web collection on statistics for biologists contains articles on many of the points above.

Software and code

Policy information about availability of computer code

Data collection	EPU 2 (Thermo Fisher)
Data analysis	cryoSPARC 3.1 (Structura Biotechnology Inc.)<br>MotionCorr2<br>Coot 0.9.4<br>Phenix 1.19.1<br>UCSF Chimera 1.15<br>UCSF ChimeraX 1.1.1<br>PyMOL (Molecular Graphics System, Version 2.1.5, Schrödinger, LLC)

For manuscripts utilizing custom algorithms or software that are central to the research but not yet described in published literature, software must be made available to editors and reviewers. We strongly encourage code deposition in a community repository (e.g. GitHub). See the Nature Research guidelines for submitting code & software for further information.

Data

Policy information about availability of data

All manuscripts must include a data availability statement. This statement should provide the following information, where applicable:

- Accession codes, unique identifiers, or web links for publicly available datasets
- A list of figures that have associated raw data
- A description of any restrictions on data availability

The atomic coordinates and the cryo-EM maps were deposited to the RCSB Protein Data Bank (PDB) and to the Electron Microscopy Data Bank (EMDB), respectively, and will become publicly available upon publication.

Field-specific reporting

Please select the one below that is the best fit for your research. If you are not sure, read the appropriate sections before making your selection.

☒ Life sciences ☐ Behavioural & social sciences ☐ Ecological, evolutionary & environmental sciences

For a reference copy of the document with all sections, see nature.com/documents/nr-reporting-summary-flat.pdf

Life sciences study design

All studies must disclose on these points even when the disclosure is negative.

Sample size	Not relevant to this study.
Data exclusions	No data were excluded.
Replication	No findings were replicated.
Randomization	Not relevant to this study.
Blinding	Not relevant to this study.

Reporting for specific materials, systems and methods

We require information from authors about some types of materials, experimental systems and methods used in many studies. Here, indicate whether each material, system or method listed is relevant to your study. If you are not sure if a list item applies to your research, read the appropriate section before selecting a response.

Materials & experimental systems

n/a	Involved in the study	
☐	☒	Antibodies
☐	☒	Eukaryotic cell lines
☒	☐	Palaeontology and archaeology
☒	☐	Animals and other organisms
☒	☐	Human research participants
☒	☐	Clinical data
☒	☐	Dual use research of concern

Methods

n/a	Involved in the study	
☒	☐	ChIP-seq
☒	☐	Flow cytometry
☒	☐	MRI-based neuroimaging

Antibodies

Antibodies used	Samples from the key steps of the purification were subjected to western blot analysis with anti-3x-FLAG antibody (Sigma, A8592). Specific proteins were detected using rabbit monoclonal antibodies against: MRPL12 (bL12m)(Proteintech; 16394-1-AP), MRPS16 (bS16m) (Proteintech; 16735-1-AP), and FLAG (Sigma, F7425); and mouse monoclonalantibodies against HA (Cell Signaling, 2367).
Validation	For Proteintech (16394-1-AP): Positive WB detected in rat brain tissue, HeLa cells, Jurkat cells;<br>For Proteintech (16735-1-AP): Positive WB detected in HEK-293 cells, Jurkat cells, HeLa cells, HepG2 cells, mouse liver, rat liver;<br>For Sigma (F7425): PRE.2.ZQ5.10000015515<br>For Sigma (A8592): PRD.1.ZQ5.10000064452<br>For Cell Signaling (2367): HA-Tag (6E2) Mouse mAb detects recombinant proteins containing the HA epitope tag. The antibody recognizes the HA-tag fused to either the amino or carboxy terminus of targeted proteins in transfected cells.

Eukaryotic cell lines

Policy information about cell lines

Cell line source(s)	HEK 293 EBNA (protein production and structure core facility at EPFL); HEK 293T
Authentication	293 c18 (ATCC® CRL-10852™), HEK 293T (ATCC® CRL-3216™)
Mycoplasma contamination	HEK 293 EBNA embryonic kidney cell were not tested for mycoplasma contamination. HEK 293T were tested and no contamination was detected.
Commonly misidentified lines (See ICLAC register)	No misidentified cell lines were used.
